# Root Caries Preventive Effect of Varnishes Containing Fluoride or Fluoride + Chlorhexidine/Cetylpyridinium Chloride In Vitro

**DOI:** 10.3390/microorganisms9040737

**Published:** 2021-04-01

**Authors:** Gerd Göstemeyer, Helen Woike, Sebastian Paris, Falk Schwendicke, Sebastian Schlafer

**Affiliations:** 1 Department of Operative and Preventive Dentistry, Charité-Universitätsmedizin Berlin, Corporate Member of Freie Universität Berlin and Humboldt-Universität zu Berlin, Aßmannshauser Straße 4-6, 14197 Berlin, Germany; helen.woike@gmail.com (H.W.); sebastian.paris@charite.de (S.P.); 2 Department of Oral Diagnostics, Digital Health and Health Services Research, Charité-Universitätsmedizin Berlin, Corporate Member of Freie Universität Berlin, Humboldt-Universität zu Berlin, Aßmannshauser Straße 4-6, 14197 Berlin, Germany; falk.Schwendicke@charite.de; 3 Section for Oral Ecology and Caries Control, Department of Dentistry and Oral Health, Aarhus University, Vennelyst Boulevard 9, 8000 Aarhus C, Denmark; sebastians@dent.au.dk

**Keywords:** biofilm model, root caries, fluoride varnish, demineralization, chlorhexidine

## Abstract

Caries preventive varnishes containing only fluoride might differ from those containing a combination of fluoride and antimicrobial components in terms of mineralization properties and their impact on the cariogenic biofilm. We compared a fluoride and a fluoride + chlorhexidine (CHX)/cetylpyridinium chloride (CPC) varnish on root caries formation in vitro. One hundred bovine root dentin samples were allocated to five groups (*n* = 20/group): (1) 7700 ppm fluoride varnish (Fluorprotector S (F)), (2) experimental placebo varnish for F (F-P), (3) 1400 ppm fluoride + 0.3% CHX/0.5% CPC varnish (Cervitec F (CF)), (4) experimental placebo varnish for CF (CF-P), (5) untreated control. Cariogenic challenge was provided using a multi-station, continuous-culture 3-species (*Streptococcus mutans* (SM), *Lactobacillus rhamnosus* (LR), *Actinomyces naeslundii* (AN)) biofilm model for 10 days. Mineral loss (ΔZ) was evaluated using transversal microradiography and bacterial counts in the biofilm assessed as colony-forming units. Fluorescence in situ hybridization (FISH) and confocal microscopy were performed to assess the three-dimensional biofilm architecture. Mean ± SD (vol% × μm) ΔZ was significantly lower for F (9133 ± 758) and CF (9835 ± 1677) compared to control (11362 ± 919) (*p* < 0.05), without significant differences between F and CF. SM counts were significantly lower and LR counts significantly higher in F- and CF-biofilms compared to control. AN counts were significantly higher in the F-biofilms than in all other groups. According to FISH, SM and LR invaded dentinal tubules only in the control-group. In the CF-group, the basal biofilm layer did not contain SM and AN. Both F and CF varnishes had similar caries-preventive effects and a considerable impact on biofilm structure and composition.

## 1. Introduction

In most industrialized countries, there is a general trend that individuals retain more teeth into higher ages [1,2]. As gingival recessions often occur during aging, the root surfaces of these retained teeth are often exposed to the oral cavity, which increases the risk for caries development [3]. Consequently, the root caries prevalence is high in older individuals and the demand for root caries management strategies can be expected to increase in the near future [4,5].

Given the difficulties in moisture control and accessibility, and with dentin being the sole adhesive substrate, restorative treatment of root caries lesions is challenging and often associated with high failure rates [6,7,8]. Cognitive decline and reduced mobility further limit the access to and provision of regular dental care for many older patients [9,10,11]. Therefore, the application of effective preventive measures is important in the management of patients at risk for root caries [12].

Chemotherapeutic approaches for caries prevention usually aim at promoting tooth mineralization using substances like fluorides or influencing the cariogenic biofilm via antimicrobial agents such as chlorhexidine (CHX) or cetylpyridinium chloride (CPC). For root caries prevention, fluorides and CHX have been shown to be clinically effective [13,14,15]. Combining fluorides with antimicrobial agents within a single varnish might lead to an improved caries prevention, assuming a synergistic effect, while the data supporting such synergism are scarce [16]. A previous study using a simple biofilm model with only *Lactobacillus rhamnosus* (LR) showed that CHX had no antibacterial and thus no caries preventive effect [17]. On the other hand, the antibacterial effect of CHX varnishes on *Streptococcus mutans* (SM), as well as the total bacterial count in artificial cariogenic biofilms is well documented [18,19,20]. It is unclear whether the use of antimicrobial components in varnishes leads to selective suppression of certain microorganisms in cariogenic biofilms and thus to a relevant change in the architecture of these biofilms. Furthermore, it is unclear to what extent such changes affect the demineralization of root dentin.

Therefore, the aim of this study was to compare the root-caries preventive effect of two commercially available varnishes containing either fluoride or fluoride + CHX/CPC, and to assess their effect on a three-species cariogenic biofilm in vitro. Our first null hypothesis was that there is no difference between fluoride and fluoride + CHX varnish regarding the prevention of biofilm-induced mineral loss. Our second null hypothesis was that there is no difference between these varnishes regarding their effect on bacterial composition and architecture of the biofilm.

## 2. Materials and Methods

### 2.1. Specimen Preparation

**Figure 1:**
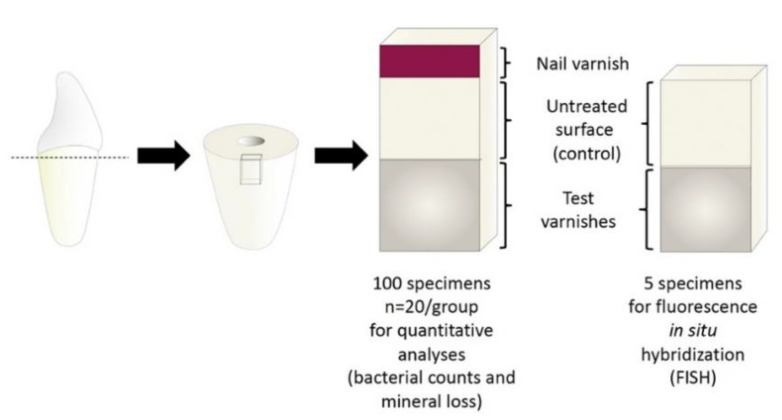
Geometry of the test specimens. All samples had an untreated control surface (3 × 3 mm) adjacent to the varnished area (3 × 3 mm). In 100 samples a reference area (1 × 3 mm) for microradiographic examination was covered with nail varnish.

The sample preparation method was performed as previously described [17,21] but the sample geometry was adapted to the requirements of the present study. Dentin specimens (20 specimens/group + 5 specimens for analysis of biofilm architecture) were prepared (2 × 3 × 7 mm; Band Saw EXAKT 300 CL; EXAKT Apparatebau, Norderstedt, Germany) from the roots of 53 bovine incisors of the second dentition, ground flat (LaboPol 25; Struers GmbH, Ballerup, Denmark/Willich, Germany) and polished (abrasive paper SiC, P 1000–4000; Buehler GmbH, Düsseldorf, Germany). Carrier bars were produced by embedding the samples (1 carrier bar/group) in acrylic resin (Technovit 4071; Heraeus Kulzer, Hanau, Germany) leaving an exposed dentin surface of 3 × 7 mm. Exposed surfaces were polished (Abrasive Paper SiC, P 1000–4000) and, in order to remove the smear layer, 1% citric acid was applied onto the dentine surfaces for 5 min, followed by rinsing with distilled water for 5 min. An area of 1 × 3 mm was covered with nail varnish (Long Lasting Nail Colour; Rival de Loop, Berlin, Germany) to serve as a reference for mineral loss assessment (Figure 1) (in the 5 samples for fluorescence in situ hybridization (FISH) this area was not covered) and the samples were sterilized (121 °C, 2.1 bar, 20 min; Tuttnauer 3870 ELV; Biomedis, Gießen, Germany). The adjacent area of 3 × 3 mm was left untreated and the remaining part of the dentin surface of 3 × 3 mm was covered with one of 4 different varnishes (two varnishes with active ingredients: fluoride (7700 ppm) varnish, 0.3% CHX + 0.5% CPC + fluoride (1400 ppm) varnish and two corresponding placebo varnishes without active ingredients) using sterile application tips (Roundtip Applicator Regular; Henry Schein, Melville, NY, USA) or left untreated in case of the control group (Table 1). All varnishes were stained with Cosmenyl Blue in order to ensure uniform application and removal of varnishes (see below).

To simulate setting under natural conditions the samples were stored in artificial saliva (defined mucin medium (DMM)) [17] for 24 h at 37 °C. The surfaces were then brushed with a soft toothbrush with a flat bristle field. Prior staining of the varnishes with cosmenyl blue ensured removal to a uniform thin film that remained on the sample surfaces. Varnishes were then rinsed with distilled water for 10 s. These procedures were performed in order to simulate removal of varnishes by mastication and oral hygiene measures. The sample surfaces were covered with sterile-filtered human saliva (collected from one 26-year-old female caries-free individual) for 2 h to induce pellicle formation and the samples were transferred to the biofilm model [22].

### 2.2. Cariogenic Challenge

A multi-station continuous-culture biofilm model firstly described by Sissons et al. [23] and later established for cultivating mono-species cariogenic biofilms [17,24,25] was used for performing the cariogenic challenge. Biofilms were grown in 5 different chambers (one carrier bar per chamber), at 100% humidity at 37 °C under aerobic conditions (Venticell 404 incubator; MMM Medcenter, Planegg, Germany). Supply of bacteria, nutrition, and artificial saliva was provided by two peristaltic multi-channel pumps (ISMATEC standard pump with MS/CA pump head; ISMATEC, Wertheim, Germany) to simulate oral conditions. Three cariogenic bacterial strains, *Lactobacillus rhamnosus* (LR) (DSM 20021), *Streptococcus mutans* (SM) (DSM 20523) and *Actinomyces naeslundii* (AN) (DSM 43013) (DSMZ—German Collection of Microorganisms and Cell Cultures, Braunschweig, Germany) were used to create cariogenic biofilms. Grow curves for each strain were generated by cultivating the bacteria in brain heart infusion (BHI; Carl Roth GmbH, Karlsruhe, Germany) + 1% sucrose at 37 °C, and the time to reach mid-log phase of the grow curve was determined (LR: 13 h, SM: 8 h, AN: 18 h) via CFU counts and measurement of optical density (OD_600_). Bacteria were grown to their respective mid-log phase in BHI + 1% sucrose, centrifuged and resuspended into fresh media. Then the three bacterial suspensions were diluted to an optical density corresponding to 9.5 × 10^8^ cells/mL.

**Figure 2:**
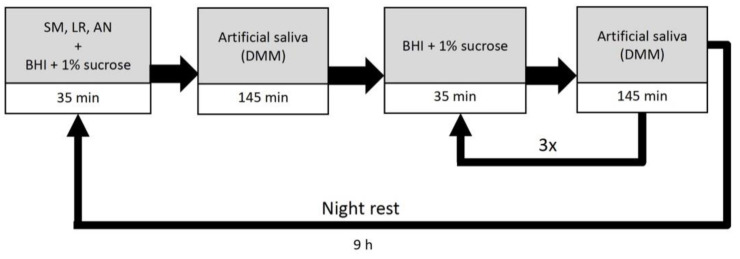
Artificial mouth model setting for the cariogenic biofilm challenge. Time for provision of the different media within a 24 h cycle is presented. Abbreviations: SM: *S. mutans*, LR: *L. rhamnosus*, AN: *A. naeslundii*, BHI: brain heart infusion, DMM: defined mucin medium.

Initially, samples were covered with the bacterial suspension for 24 h under static conditions to allow for bacterial adhesion to the sample surfaces. During the cariogenic challenge, the samples were subjected to a daily cycle of provision of different media for 10 days (Figure 2). At the beginning of a daily cycle, samples were inoculated with 50 mL/carrier bar of bacterial suspension for 35 min, followed by provision of 50 mL DMM for 145 min. To simulate nutrition supply, samples were then provided with 50 mL BHI + 1% sucrose solution for 35 min followed by DMM for 145 min. This cycle was repeated 4 times before simulating a night rest for 9 h without media provision. After 10 days samples were removed and analyzed.

### 2.3. Assessment of Mineral Loss

Transvers microradiography was used to assess mineral loss (ΔZ (vol% × μm)) and lesion depth (LD (µm)). Detailed descriptions of this methodology can be found elsewhere [26]. The samples were cut perpendicularly to the test surface (Band Saw EXAKT 300 CL) and ground flat to 100 ± 10 μm (EXAKT Mikroschleifsystem 400 CS; EXAKT; abrasive paper WS flex 18 C; SiC; P 1200–4000; Hermes Schleifmittel, Hamburg, Germany). Samples were imbibed in 99% ethylene glycol (Sigma Aldrich, Steinheim, Germany) and transferred to the X-ray source. Microradiographs were obtained using a nickel-filtered copper X-ray source (X-ray tube PW2213/20; Panalytical, Kassel, Germany; x-ray generator PW 1730/10; Philips, Eindhoven, The Netherlands) operating at 20 kV and 10 mA with an exposure time of 10 s. Films (Fine 71337; Fujifilm, Tokyo, Japan) were developed according to manufacturer’s recommendation. A digital-image-analysing system (CFW 1312M; Scion, Frederick, MD, USA), interfaced with a universal microscope (CCD-video camera module XC 77 CE; Sony, Tokyo) and dedicated software (TMR for Windows; Version 2.0.27.2; Inspektor Research, Amsterdam, The Netherlands) was used to analyze the lesions.

### 2.4. Assessment of Bacterial Counts

Three biofilm samples were collected from each effect surface using sterile standardized plastic spatulas (DC Composite Einwegspatel; DC DentalCentral, Hannover, Germany), in order to ensure removal of similar amounts of biofilm per sample. Biofilm samples were transferred to 1 mL NaCl (0.9%), vortex mixed for 10 s and plated on different selective agars in various dilutions (10^5^–10^7^): De Man, Rogosa and Sharpe (MRS) agar [22] to assess LR counts, Mitis Salivarius Agar [23] containing Bacitracin to assess SM counts and Cadmium Sulfate-Fluoride-Acridine Trypticase (CFAT) agar [24] to assess AN counts. Agar plates were incubated for 48 h at 37 °C and 5% CO_2_ (CO_2_ Incubator, Heraeus Kulzer, Hanau, Germany). After cultivation, colony forming units (CFU) per ml were enumerated.

### 2.5. Preparation of Samples for Fluorescence In Situ Hybridization

Five tooth specimens were subjected to FISH after biofilm growth to study biofilm architecture. Samples were processed as described previously [27]. In brief, specimens were fixed for 16 h in 3% paraformaldehyde and then washed three times in PBS for 20 min. Thereafter ethanol dehydration was performed in 50%, 60%, 70%, 80%, 90% and 100% ethanol-PBS (vol/vol) for one h each. Afterwards the specimens were infiltrated twice for 3 h with cold polymerizing resin (Technovit 8100; Heraeus Kulzer, Hanau, Germany). During the first 10 min of both infiltration steps, samples were placed in a vacuum chamber (Exsikkator; Kartell S.p.A. LABWARE Division, Noviglio, Italy). Specimens were then placed in polyethylene embedding molds (Flat Bottom Embedding Capsules; Electron Microscopy Sciences, Hatfield, PA, USA), and embedded overnight on ice. All procedures were performed at 4 °C. From the embedded specimens, slices of 1 mm were sectioned with a saw microtome (Ernst Leitz, Wetzlar, Germany) and decalcified in 17% EDTA for 18 days. Successful decalcification was verified by digital x-ray analysis, after which the slices were re-imbedded in resin (Technovit 8100). Finally, thin sections of <2 µm were produced on an ultramicrotome (Ultracut E; Reichert Jung Optische Werke, Wien, Austria) and mounted on coated glass slides (Polysine; Menzel-Gläser, Braunschweig, Germany).

### 2.6. FISH

SM and AN were detected with the probes STR405 (5′-TAG CCG TCC CTT TCT GGT-3′) and ACT476 (5′-ATC CAG CTA CCG TCA ACC-3′), respectively. The broad range bacterial probe EUB338 (5′-GCT GCC TCC CGT AGG AGT-3′) was employed to visualize all three bacteria. Hence, LR was only targeted by EUB338. All probes were 5′-end-labelled (IBA, Göttingen, Germany), STR405 with Alexa488, ACT476 with Cy3, and EUB338 with Cy5. The FISH protocol is described in detail elsewhere [28]. In brief, cells were lysozyme-treated for 9 min at 37 °C (70 U µL^−1^; Sigma, Brøndby, Denmark) and then hybridized in a humid chamber at 30% formamide and 46 °C for 2 h. A stringency wash was performed at 48 °C, after which a cover glass was mounted with Citifluor AF1 (Citifluor, Hatfield, PA, USA). In all experiments, fixed cells of *S. mitis* SK24, *S. downei* HG594, *A. naeslundii* AK6 and *L. rhamnosus* DSM 20021 served as positive and negative controls for the employed probes (Figure S1).

### 2.7. Confocal Laser Scanning Microscopy

The samples were analyzed as described previously [29], using a confocal laser scanning microscope (Zeiss LSM 700, Jena, Germany) equipped with a 63× objective (alpha Plan-Apochromat, Zeiss). Alexa488 was excited at 488 nm and detected with a 640 nm short pass filter; Cy3 was excited at 543 nm and detected with a 560 nm long pass filter. Cy5 was excited at 639 nm and detected with a 640 nm long pass filter. The different dyes were imaged sequentially to avoid cross talk between channels. Images were acquired with the pinhole set to 1 Airy unit, a pixel dwell time of 2.71 μs, line average 4 and a size of 1192 × 1192 pixels.

### 2.8. Statistical Analysis

Statistical analysis was performed with SPSS 25 (IBM, Armonk, NY, USA). Shapiro-Wilk and Levene tests, respectively, showed normal distribution and homogeneity of variance for all assessed parameters. Differences in ΔZ and bacterial counts between groups were analyzed using one-way analysis of variance (ANOVA). Post-hoc Tukey’s HSD test was used to assess significant differences between groups. The level of significance was set at α = 0.05.

## 3. Results

### 3.1. TMR

**Figure 3:**
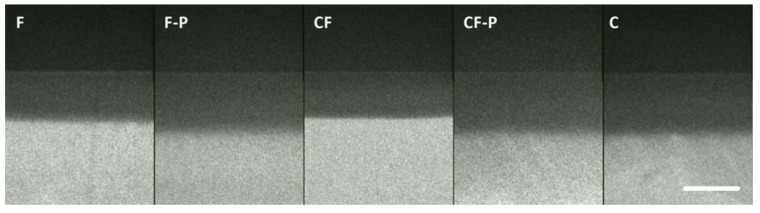
Representative microradiographs of the biofilm induced caries lesions for the different test groups. Abbreviations: F: fluoride varnish, F-P: placebo varnish for F, CF: fluoride + CHX varnish, CF-P: placebo varnish for CF, C: control group. Scale bar = 300 μm.

**Figure 4:**
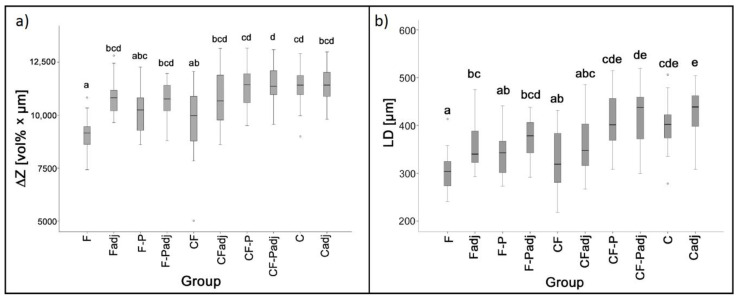
Mineral loss (ΔZ) (**a**) and lesion depth (**b**) for the different test groups. Medians, quartiles and extreme values are given. Groups denoted by same letters do not differ significantly (ANOVA/Tukey HSD). Abbreviations: F: fluoride varnish, F-P: placebo varnish for F, CF: fluoride + CHX varnish, CF-P: placebo varnish for CF, C: control group, adj: estimation of ΔZ adjacent to varnished area.

Representative microradiographs of the different groups can be found in Figure 3. F and CF-covered surfaces showed the lowest mineral loss (Mean ± SD: 9133 ± 758 vol% × μm and 9835 ± 1677 vol% × μm) and lesion depth (305 ± 42 μm and 330 ± 60 μm), respectively. For both varnishes, mineral loss was significantly lower than in the control group (11363 ± 919) (F: *p* < 0.001, CF: *p* = 0.008) and in the CF-P group (11310 ± 983) (F: *p* < 0.001, CF: *p* = 0.012), with no significant difference between F and CF (*p* = 0.771). The difference between both varnishes (F and CF) and F-P (10009 ± 220) did not reach the level of statistical significance (F: *p* = 0.35, CF: *p* = 1). Both varnishes did not protect adjacent areas from mineral loss. Differences in LD between the different groups followed the trend as for the ΔZ values (Figure 4).

### 3.2. Bacterial Counts

**Figure 5:**
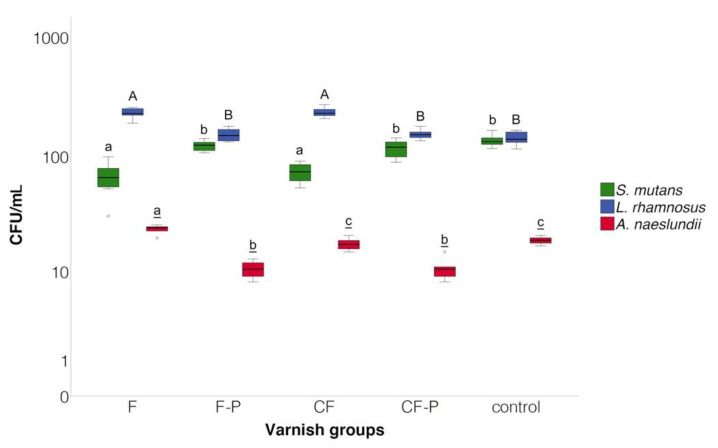
Bacterial counts (CFU/mL) at 10^5^ dilution of the different bacteria strains. Medians, quartiles and extreme values are given. Box plots denoted with the same letter (SM: lower case letters, LR: upper case letters, AN: underlined letters) do not differ significantly (ANOVA/Tukey HSD); Abbreviations: SM: *S. mutans*, LR: *L. rhamnosus*, AN: *A. naeslundii*, F: Fluoride varnish, F-P: Placebo varnish for F, CF: Fluoride + CHX varnish, CF-P: Placebo varnish for CF, C: Control group.

Mean ± SD (107 CFU/mL) SM counts were significantly lower in the F (0.67 ± 0.19) and CF (0.73 ± 0.13) groups compared to all other groups (*p* < 0.05). LR counts were significantly higher in the F (2.31 ± 0.20) and CF (2.33 ± 0.20) groups compared to all other groups and did not differ significantly between placebo varnishes and control group (1.43 ± 0.19). For AN, CFU counts were significantly higher in the F (0.24 ± 0.02) group compared to all other groups (*p* > 0.05). AN counts did not differ significantly between the CF (0.18 ± 0.02) and the control group (0.19 ± 0.01) (*p* = 0.49), and CFUs in the placebo varnish groups were significantly lower than in all other groups (Figure 5).

### 3.3. FISH

**Figure 6:**
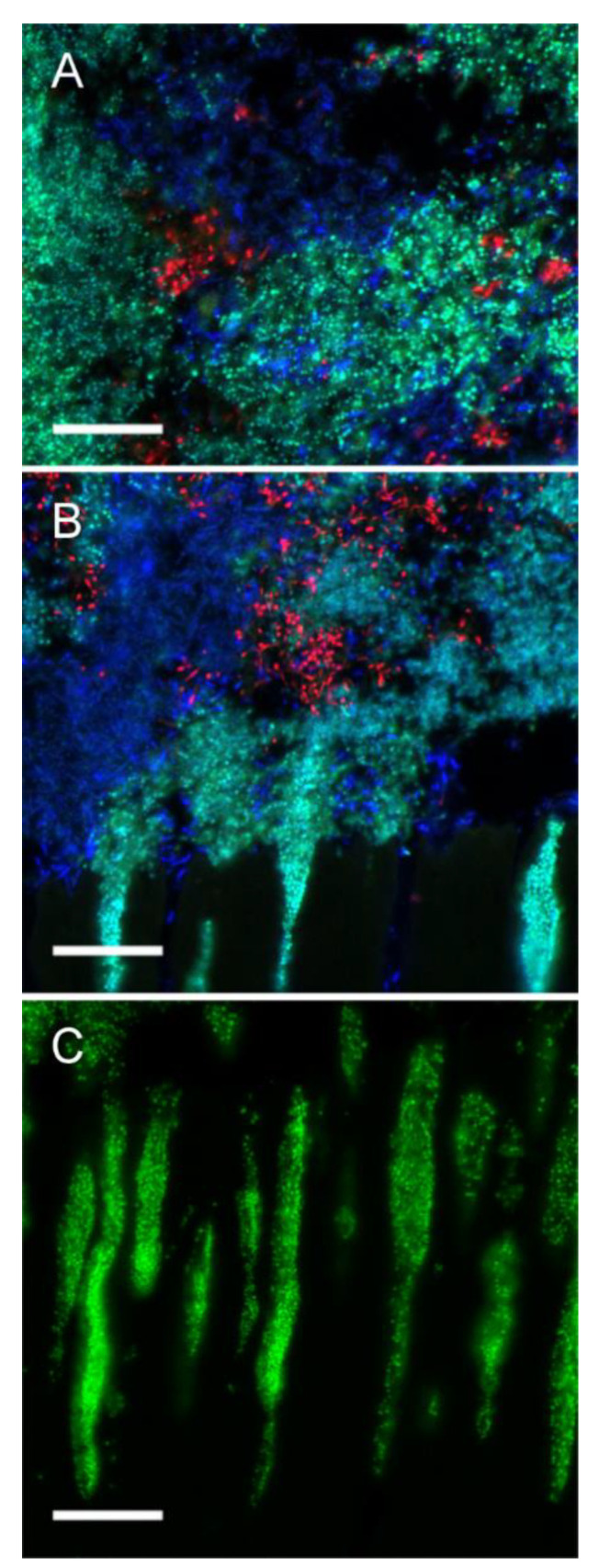
Fluorescence in situ hybridization (FISH) of control biofilms. FISH demonstrated the presence of relatively thick (>200 µm) biofilms comprising all three employed bacterial strains. Control biofilms were dominated by large clusters of *S. mutans* (SM) (green/turquoise) and *L. rhamnosus* (LR) (blue), with smaller colonies of *A. naeslundii* (AN) (red) (**A**,**B**). Both LR (**B**) and SM (**B**,**C**), but not AN penetrated dentinal tubules, with a penetration depth of up to 100 µm in some areas (**B**,**C**). Bars = 20 µm.

**Figure 7:**
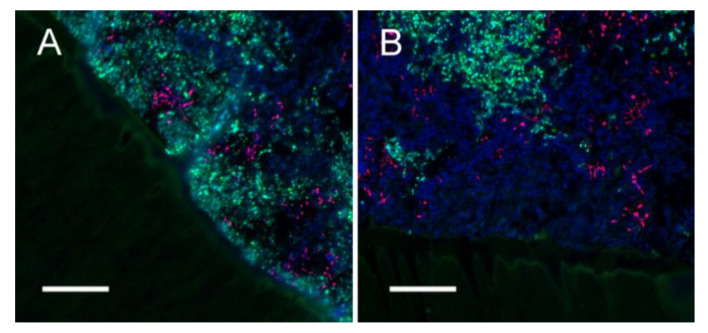
Fluorescence in situ hybridization (FISH) of biofilms treated with F (**A**) or CF (**B**). For both treatment groups, thick biofilms containing all employed strains were observed. Biofilms were dominated by *L. rhamnosus* (LR) (blue) and *S. mutans* (SM) (green/turquoise), with small, interspersed clusters of *A. naeslundii* (AN) (red). Interestingly, the basal layer of the biofilm on specimens treated with fluoride + CHX varnish contained only LR (**B**). The biofilms were firmly attached to the underlying dentin, but the layer of varnish prevented any bacterial penetration of dentinal tubules.

SM and AN were reliably detected by FISH with the genus specific probes STR405 and ACT476. All strains were targeted by EUB338, which allowed identifying LR in the three-species model (Figure S1). All examined tooth specimens showed thick biofilms (>200 μm) including all three employed bacterial strains (Figure 6A). Biofilms were dominated by large clusters of SM and LR, interspersed with minor colonies of AN. In the control samples, SM and LR invaded dentinal tubules in all areas of the biofilm, to a depth of up to 100 μm (Figure 6B,C). In samples treated with F or CF, biofilm thickness was similar to control samples, but both varnishes prevented bacterial invasion of dentinal tubules (Figure 7). Interestingly, only few cells of SM were observed in basal layers of the biofilm on the specimens treated with CF (Figure 7B).

## 4. Discussion

Given the high prevalence of root caries in the older population, there is a high demand for effective root caries preventive agents. Application of caries preventive varnishes containing a combination of fluoride and antimicrobial substances might be promising for this purpose. Within this study, employing a three-species in vitro biofilm model, we did not find any significant difference between two commercially available varnishes containing F or CF in prevention of mineral loss. Our first null hypothesis was thus accepted. We furthermore found only minor differences in biofilm composition and architecture between both varnishes. Therefore, our second null hypothesis was partially accepted.

F and CF exhibited similar caries preventive properties in our biofilm model. This is in line with the results of an in-situ study where application of a 1:1 mixture of a fluoride varnish and a CHX varnish resulted in a similar mineral loss as fluoride varnish alone [30]. However, in our investigation the varnishes differed considerably in fluoride concentrations (F: 7700 ppm, CP: 1500 ppm). We used the varnishes in their commercially available form, as it was not possible for the manufacturer to produce varnishes with equal fluoride concentrations using the respective carrier substances for F and CP varnishes. With the caries preventive effect of fluoride being dose dependent [31,32], it can be speculated that using a fluoride concentration in the CF-group similar to the F group might have resulted in a more pronounced caries preventive effect.

The varnishes also varied in the composition of their carrier substances (i.e., inactive ingredients). The inactive components of the varnish serve to control the release kinetics of the active ingredients and thus control the concentration of the active ingredients on the tooth surface and in saliva. Therefore, in addition to the concentration of the active ingredients of the varnish, the release kinetics of the inactive ingredients also determine its caries preventive effect [33]. The inactive ingredients of the varnish, however, have to be tailored according to the type of active substances used, which is why the composition of inactive ingredients between F and CF also differs. Due to the different compositions and fluoride concentrations, it is therefore not possible to evaluate, on the basis of our data, whether the addition of CHX to a fluoride varnish has an additional caries-preventive effect. However, our study still provides useful insights on the effect of the different tested varnishes on mineral loss and the biofilm composition and architecture.

Interestingly, application of F-P resulted in lower mineral loss (albeit not significant) than control treatment, with no significant difference compared to F-varnish. In contrast, CF-P had no effect on mineral loss. The tendency towards a caries preventive effect of F-P might be explained by the formation of a protective mechanical barrier (i.e., sealing) on the dentin surface or within the dentinal tubules. As CF-P differed in composition from F-P, it can be speculated that the sealing ability of the carrier substance of a varnish contributes to its caries preventive effect. Further studies should investigate by which parameters, such as dissolution and adhesion behavior, a potential caries preventive effect can be modulated by the composition of the carrier substance of varnishes.

We further found that none of the varnishes protected adjacent areas from mineral loss. This is in contrast to previous findings where CHX had a peripheral caries-preventive effect [30]. As we aimed to simulate the removal of varnishes from the root surface due to mastication and oral hygiene measures, we reduced the thickness of the varnishes 24 h after application, leaving a homogeneous thin film on the sample surfaces (see Figure 7). It may be that the remaining amount of varnish was not sufficient to release enough of the active ingredients to have an effect on adjacent areas. The amount of remaining varnish on the root surfaces might have a significant influence on the caries preventive effect not only on the applied area but also on adjacent areas. Clinically, it is likely that varnishes are quickly being removed from root surfaces that are well accessible to mastication and oral hygiene measures but might remain for longer in sheltered areas [34]. However, due to this high clinical variability, different modes of removal of the varnishes cannot be reliably simulated in vitro.

Interestingly, F and CF varnishes had quite similar effects on bacterial counts. In both groups, SM counts were significantly reduced and LR counts significantly increased, as compared to the placebo varnishes and the control group. The antibacterial effect of CHX on SM is well known [15,16] and has also been demonstrated clinically for fluoride varnishes containing CHX [35,36]. CPC also has an antibacterial effect against oral *Streptococci*, but to a lesser extent compared to CHX [37]. An antibacterial effect against SM for ammonium fluoride, which is the active ingredient of F, was also found in an in vitro study [33]. However, these findings were not confirmed in situ, where neither fluoride nor fluoride + CHX varnishes had an effect on SM and *Lactobacilli* counts on dentin samples after being exposed to the oral environment for three weeks [30]. This might indicate that the antibacterial effect of the varnishes decreases quickly over time. In line with our results, no antibacterial effect of CHX against LR in biofilms on root dentin could be found in other in vitro studies [17,38]. The increased LR counts in the F and CF groups might indicate that LR bacteria have replaced SM bacteria in the biofilms. Furthermore, AN counts were significantly higher in the F compared to all other groups, while they did not differ significantly between the CF and control group. The latter is in line with results from previous clinical studies, where application of a fluoride and a CHX varnish did not result in a decrease of *A. viscosus/naeslundii* numbers [35,39]. Overall, it can be assumed that the antibacterial activity of F and CF varnishes led to a selective suppression of SM whilst allowing LR numbers, and for F also AN numbers, to increase. However, as the differences between the groups were relatively small (all approximately within one log), it is questionable whether the reduction in bacterial counts leads to a significant clinical effect.

The intraoral use of CHX, however, is increasingly viewed critically against the background of resistance developments [40]. For other clinical applications, critical resistance developments that were associated with the use of CHX have already been demonstrated [41]. The emergence of variants with reduced susceptibility following exposure to CHX has also been demonstrated for *S. mutans*. However, this was associated with a reduced ability to compete with other commensal streptococci within oral biofilms and an increased susceptibility to other antimicrobials [42].

According to our FISH analysis, there were no differences in thickness and overall architecture of the biofilms between F and CF varnishes and the control group. All biofilms showed a typical structure for supragingival biofilms, with vertical protuberances, dense bacterial clusters and bacteria-free voids, likely filled with extracellular polymeric substances [43]. As observed in in situ-grown biofilms, colonies of AN were branched and interwoven with the other two species [28]. In the varnish groups, there was no bacterial penetration into the dentin tubules, whereas dentin tubules in the control group were infiltrated with SM and, to a lesser extent, LR. This might be the result of varnish material remaining on the dentin surface or within the dentin tubules. It could be demonstrated that caries preventive varnishes are able to penetrate into dentin tubules [44]. Hence, as mentioned above, remaining material may have acted as a depot for the release of active ingredients, or simply as a mechanical barrier. Theoretically, both mechanisms should lead to a caries preventive effect. However, for capacity reasons, biofilms on samples with placebo varnishes were not assessed via FISH in our investigation. Therefore, we cannot clearly determine which effect dominates. The finding that there were no significant differences in mineral loss between F and CF varnishes and the F placebo varnish indicates that the effect of a mechanical barrier seems to have a caries preventive effect. The further finding that the basal layer of the biofilms in the CF group did not contain SM indicates that there may at least be a localized selective effect of the CHX component on streptococci. As this is, to the best knowledge of the authors, the first study to analyze the architecture of biofilms on varnished teeth using FISH, our findings cannot be confirmed or refused by other investigations. Further structural analyses using FISH seem promising to gain deeper insight into the effect of caries-preventive varnishes on cariogenic biofilms.

Our study has a number of limitations. First, the employed biofilm model was simple as it contained only three bacterial species and was highly cariogenic. Oral biofilms are more complex and demineralization dynamics produced by natural biofilms certainly differ from those in our study. However, a realistic modelling of cariogenic biofilms in vitro is almost impossible [45] and would still have limited generalizability due to intra- and interindividual variability [45]. With the selection of the different strains, we wanted to replicate the biodiversity as closely as possible within the limits of the in vitro model in order to be able to investigate possible changes in biofilm composition and architecture due to different sensitivities of the bacteria to CHX or CPC. All three bacterial strains studied play a role in the development of root caries [46,47]. Interestingly, LR is also used as a probiotic. However, this bacterium has been shown to be highly cariogenic, which calls into question its clinical utility as a probiotic strain [24]. Secondly, the additional effect of daily use of fluoridated dentifrices was not evaluated, as we aimed to clearly depict the effect of the varnishes. The simulation of the additional use of fluoridated dentifrices, although clinically relevant, could have distorted our results. Moreover, in view of the fact that in our experiment we simulated cariogenic conditions in elderly patients who do not always receive adequate oral hygiene measures, our approach seems justified. Thirdly, ground and polished bovine roots were used for sample preparation. Since there are only minor differences in demineralization behavior between human and bovine root dentin [48], it should be justified to extrapolate the findings of the present study to human root dentin. We acknowledge that the polished, flat surfaces used in our study deviate from the natural anatomy of the root surface. This sample preparation, however, was necessary in order to perform a reliable micro-radiographic evaluation. Fourth, we removed the varnishes after 24 h leaving a thin varnish layer behind. This was done because we wanted to avoid the varnishes to primarily act by forming a mechanical caries protective barrier on the tooth surface and simultaneously allow the measurement of possible effects of active substances released from the varnish residues. Since clinically the varnishes are removed to different extents depending on the localization on the tooth, our approach simulates only one of many possible clinical situations.

Overall, our results neither support nor refuse the use of varnishes containing fluoride and CHX/CPC in combination. Based on the results of the present study and within its limitations, F and CF varnishes have similar effects on root caries prevention and the overall bacterial composition of the cariogenic biofilm. Assessment of the biofilm architecture indicated that varnishes prevented dentine tubules from bacterial infiltration and the antimicrobial component of CF led to an elimination of SM in basal layers of the biofilm. Further studies should assess the relevance of these effects for root caries prevention.

## Figures and Tables

**Table 1:** Brand names, active ingredients and batch numbers of the test groups.

Group	Name (Manufacturer)	Active Ingredient (s)	Inactive Ingredients (wt.-%)	Batch No.
F	Fluor Protector S^®^ (Ivoclar Vivadent AG)	7700 ppm ammonium fluoride	Ethanol/water (73.4), polymer, additive (25.0), saccharin, mint flavoring (0.1)	X14474
F-P	Placebo varnish for F (Ivoclar Vivadent AG)	Same ingredients as F except for ammonium fluoride		Experimental placebo varnish provided by the manufacturer exclusively for the purpose of this study
CF	Cervitec F^®^ (Ivoclar Vivadent AG)	0.3% chlorhexidine, 0.5% cetylpyridinium chloride, 1400 ppm ammonium fluoride	Ethanol/water (80–90), Vinyl acetate/crotonic acid copolymer (8–12), saccharin, mint flavoring (<1)	X15712
CF-P	Placebo varnish for CF (Ivoclar Vivadent AG)	Same ingredients as CF except for CHX and ammonium fluoride		Experimental placebo varnish provided by the manufacturer exclusively for the purpose of this study
C	Untreated control	-		-

## Data Availability

No new data were created or analyzed in this study. Data sharing is not applicable to this article.

## Supplementary Materials

The following are available online at https://www.mdpi.com/article/10.3390/microorganisms9040737/s1, Figure S1: Assessment of probe specificity.
