# Supplementary material for: Root Caries Preventive Effect of Varnishes Containing Fluoride or Fluoride + Chlorhexidine/Cetylpyridinium Chloride In Vitro
Source: Microorganisms. 2021 Apr 1;9(4):737. doi: 10.3390/microorganisms9040737 (PMC8065905; doi:10.3390/microorganisms9040737)
Supplement: Supplementary file 1 [file microorganisms-09-00737-s001.pdf]

Supplementary

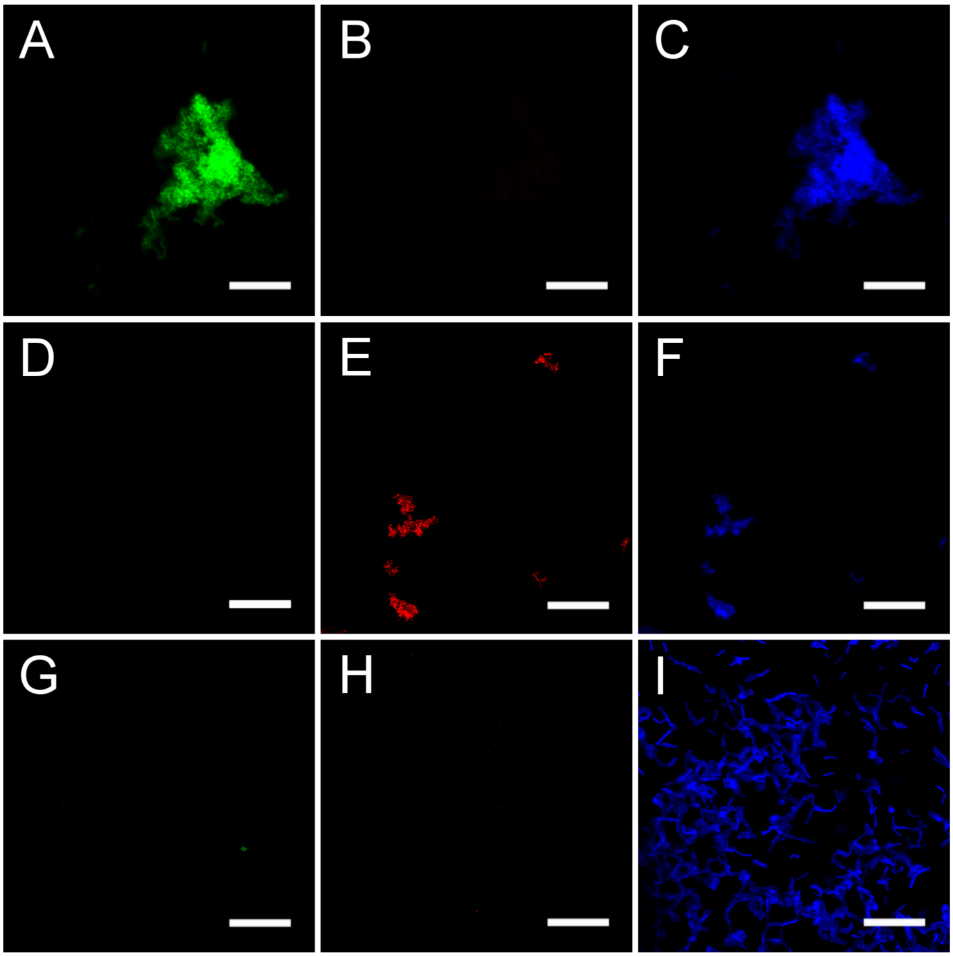

Figure S1. Assessment of probe specificity. Fluorescence in situ hybridization was performed with the probes STR405 (shown in green), targeting Streptococci, ACT476 (red), targeting *Actinomyces* spp. and EUB338 (blue), targeting all bacteria in the biofilms. Fixed cells of *Streptococcus mitis*, *AN* and *LR* served as positive and negative controls for the three probes. *S. mitis* was stained by STR405 (A) and EUB338 (C), but not by ACT476 (B). *AN* was not detected by STR405 (D), but stained by ACT476 (E) and EUB338 (F). *LR* was not visualized by any of the genus-specific probes (G and H), but by EUB338 (I). Bars = 20 μm.
